# Estimating population sizes to evaluate progress in conservation of endangered golden lion tamarins (*Leontopithecus rosalia*)

**DOI:** 10.1371/journal.pone.0216664

**Published:** 2019-06-05

**Authors:** Carlos R. Ruiz-Miranda, Marcio M. de Morais, Lou Ann Dietz, Brenda Rocha Alexandre, Andréia F. Martins, Luís Paulo Ferraz, Jennifer Mickelberg, Sarah J. Hankerson, James M. Dietz

**Affiliations:** 1 Laboratório de Ciências Ambientais, Universidade Estadual do Norte Fluminense, Campos dos Goytacazes, Rio de Janeiro, Brazil; 2 Associação Mico-Leão-Dourado, Silva Jardim, Rio de Janeiro, Brazil; 3 Save the Golden Lion Tamarin, Silver Spring, Maryland, United States of America; 4 Programa de Pós‐Graduação em Ecologia, Departamento de Ecologia, Universidade Federal do Rio de Janeiro, Rio de Janeiro, Brazil; 5 Instituto de Geociências, Universidade Federal Fluminense, Campus Praia Vermelha, Niterói, Rio de Janeiro, Brazil; 6 Zoo Atlanta, Atlanta, Georgia, United States of America; 7 Department of Psychology, University of St. Thomas, St. Paul, Minnesota, United States of America; National Zoological Park, UNITED STATES

## Abstract

Efforts to reverse the decline of endangered golden lion tamarin monkeys have been relatively successful because the Brazilian organization dedicated to the species’ conservation (Associação Mico-Leão-Dourado, AMLD) relies on science-based computer modeling to determine the number of tamarins necessary to achieve demographic and genetic goals, and a process of strategic planning to achieve those goals. Accurate estimates of the numbers of tamarins in forest fragments are essential to evaluate progress in achieving goals and adapt strategies as necessary. In this report we present the results of a new method to survey the number of tamarins in the wild, a modification of the point transect with lures procedure. Using this method, we estimate that in 2014 there were approximately 3,700 golden lion tamarins in 41,400 hectares of Atlantic Forest. Of these, 59% are from remnant wild populations, 34% are descendants of captive-born reintroduced animals and 7% are descendants of wild translocated groups. The number of tamarins and amount of forest estimated in this survey exceeded values necessary to meet AMLD’s definition of a viable population, determined to be 2,000 tamarins in 25,000 hectares of connected and protected forest. However, the seven forest blocks and their tamarin populations are not yet adequately connected and protected. AMLD’s strategic plan to achieve a viable population of golden lion tamarins includes 12 strategies that mitigate these and other threats or contribute directly to the conservation goal. The point transect with lures survey method provides a way to evaluate progress in achieving that goal and adapt strategies as appropriate.

## Introduction

**Fig 1:**
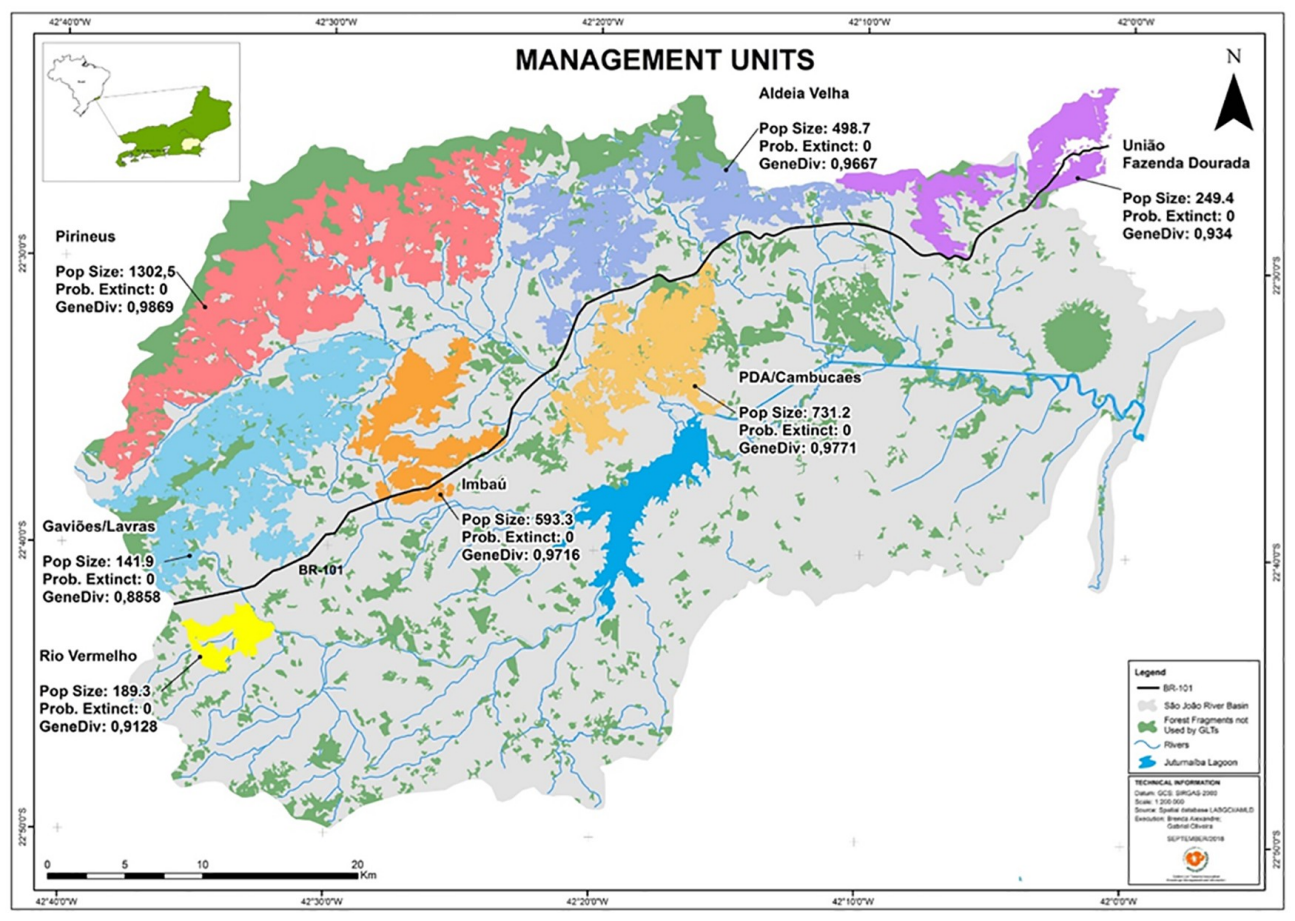
Map of current geographic distribution of golden lion tamarins. Map showing the current geographic distribution of golden lion tamarins. Colored polygons represent blocks of connected forest fragments (as we understood connectivity in 2014) which we refer to as management units with GLT populations. Dark blue is a body of water, green shows forest fragments not used by GLTs. Estimates of GLT population sizes and forest area are shown for each management unit.

In contrast with the dismal predictions for many endangered species [1, 2] the recovery of golden lion tamarins (*Leontopithecus rosalia*; GLTs) in Brazil’s Atlantic coastal rainforest is seen as a conservation success story [3, 4]. Golden lion tamarins are small arboreal primates found only in lowland Atlantic Forest, Rio de Janeiro state, Brazil. Centuries of deforestation for timber and charcoal production, agriculture and cattle ranching, followed by urban expansion, reduced the tamarin’s lowland-forest habitat to ca. 0.4% of its original area [5]—all fragmented into small and isolated forest islands surrounded by cattle pasture [6]. Golden lion tamarins are found in 7 forest fragments (as we understood them in 2014) over an area of ca. 4,500 km^2^ of lowland Atlantic coastal rainforest, most of which is in the São João river basin (2,150 km^2^, of which 33% is forest; Fig 1). Each fragment has limited or no forest connection with other fragments. We refer to these fragments as management units (MUs).

In the 1960s and 1970s, the in situ GLT population was estimated at a few hundred individuals [7–9]. In 1983, the Smithsonian’s National Zoo began long-term conservation initiatives in Rio de Janeiro, Brazil, including studies of the demography and social ecology of GLTs, reintroduction of zoo-born GLTs, relocation of rescued GLTs and community environmental education [10–15]. In 1992, the National Zoo’s conservation program was transformed into the Associação Mico-Leão-Dourado (golden lion tamarin association; AMLD), a Brazilian non-governmental organization whose mission is to keep GLTs from extinction in Brazil’s Atlantic Forest. As a result of early efforts, GLT populations rebounded. AMLD’s three decades of environmental education work contributed to creation of a conservation ethic in the region and the halting of illegal deforestation and capture of GLTs for the pet trade [16–18]. In 2003, the conservation status of GLTs was changed from “critically endangered” to “endangered” [19]. Rationale for the change included increases in population size and area of occupancy [12, 19, 20]. In 2017, GLTs were found in all the largest remaining forest fragments in the São João river basin—seven municipalities located 80 km northeast of Rio de Janeiro city with over 12 million people.

The design of AMLD’s strategic plan for 2016–2025 follows the Open Standards for the Practice of Conservation [21] and is focused on achieving desired impacts on two conservation targets: the GLT species and its Atlantic Forest habitat. Summarizing, the Open Standards are recommendations for best practices in adaptive management for biodiversity conservation, combining conservation action and research in a continuous cycle which includes setting achievable goals, identifying threats to achieving those goals, designing strategies and implementing actions to mitigate threats, monitoring results and adapting the strategic plan as appropriate to improve results. AMLD documents this process in Miradi adaptive management software [22]. Miradi allows nature conservation practitioners to design, manage, monitor, and learn from their projects to more effectively meet their conservation goals. Miradi contains tools to visualize complex relationships in the current situation using multidimensional diagrams called conceptual models [23, 24] and diagrams of their theories of change called results chains. Miradi also facilitates development of detailed work plans, budgets and recording of monitoring results as well as adaptation for subsequent cycles and producing reports for sharing learning.

### Measuring the current and desired future conservation status of golden lion tamarins

AMLD’s conservation goal has always been to ensure the existence a block of connected habitat large enough to hold a population of GLTs that would be demographically and genetically viable in the long term. To estimate the size of a minimum viable population for GLTs [25, 26] AMLD and collaborators used VORTEX simulation software (version 9.99; [27, 28]), a computer model that follows the fate of individuals based on parameters of the population provided by the user. After running many iterations, VORTEX reports the probability of extinction of the starting population and the percent retention of genetic diversity after a predetermined period of time, e.g. 100 years. We used VORTEX to estimate the size of a GLT population necessary to meet these demographic and genetic criteria: 0% probability of extinction and 98% retention of genetic diversity over a period of 100 years. We also used VORTEX to evaluate the current status of each GLT population and of the metapopulation. Results from those simulations are also used to determine management needs for each population, evaluate the efficacy of methods in AMLD’s strategic plan and adapt as necessary.

To measure progress in achieving our goal, a viable population of GLTs as we define it, we must have reliable estimates of a key indicator: GLT population sizes in each isolated forest fragment. AMLD’s previous estimates of GLT population sizes were made by extrapolating GLT densities from long-term studies to areas where GLTs were not studied. Long-term data on territory sizes and group compositions in Poço das Antas Biological Reserve allowed calculation of GLT densities under high and low predation [29]. Population sizes in forest fragments for which we did not have complete counts of GLTs were estimated by multiplying the minimum observed GLT density estimate from Poço das Antas (0.073 GLTs/ha; [29]) by forest fragment areas calculated from progressively higher-resolution satellite images. In 2013, this calculation yielded an estimate of ca. 3,200 GLTs in the wild. However, this method relied on assumptions that we could not test, e.g. that GLT densities in fragments with no monitored GLT groups were similar to those in monitored groups in Poço das Antas Reserve.

To secure more reliable estimates of GLT population sizes in areas where monitoring is limited, and thus improve conservation management, we adapted the “point transect with lures” procedure (detailed in [30]) used to census cotton-top tamarins (*Saguinus oedipus*) in Colombia [31, 32]. We refined this method by first conducting a field experiment to estimate the detection function (see Methods) and then applied the refined survey methods throughout the geographic distribution of GLTs. In this report we present the results of that comprehensive baseline census and discuss how those results are used to evaluate progress in AMLD’s comprehensive strategic plan for conservation of the species.

## Methods

The study followed the ethical guidelines of the International Primatological Society and complied with all applicable Brazilian laws. Research and management permits were obtained from the Brazilian government agencies (Instituto Chico Mendes para Conservação da Biodiversidade): permit number 17409-(09–12). Previous research on the Poço das Antas population complied with the guidelines of the University of Maryland Animal Care and Use Committee.

### Estimating the detection function using experimental playbacks of GLT vocalizations

The accuracy of the point transect with lures method depends on the detection function, the probability of detecting an animal as a function of its distance from the “lure” point, using logistic regression. Because GLTs respond to amplified recordings of long-distance vocalizations of other GLTs, i.e. long calls, [12, 33, 34], we used playbacks of these vocalizations as lures. The detection function is directly related to how the animals respond to the playbacks and to the percent of the group that can be detected [31, 32]. In order to estimate the detection function for GLTs, we carried out a preliminary study between August 2013 and November 2013 in forest fragments where radio-tagged tamarins were monitored weekly. Ten groups with radio collars and individually marked GLTs were selected for the experiment. Five groups were in two large forest fragments (Poço das Antas Biological Reserve, 3,606 ha forest; União Biological Reserve, 5,584 ha forest) and five groups were in three small fragments (each less than 60 ha). All individuals in the 10 groups were habituated to the presence of human observers. Two observers with decades of experience collecting data on GLT behavior located the social group and recorded the number of individuals, sex, age class, location and behavioral data for 10 min. Then one of the observers (hereafter the surveyor) distanced him/herself from the tamarin group, while the other (the observer) remained with the group. The surveyor used playback equipment (digital recorder and speaker) to play GLT vocalizations at 30, 60, 90 and 120 m from the estimated center of the group. We selected those distances based on previous research on the attenuation of GLT calls in the forest [35, 36]. The calls used for playbacks were obtained from social groups unknown to the study groups. The playback consisted of male and female long calls separated by 3 seconds of silence played twice per playback point. Calls from both sexes were used to simulate a social group challenge as previous studies reported that responses are specific to the sex of the caller [34]. The behavioral data collected by the observer included recording immediately after playback the orientation or movement toward the speaker of the reproductive male, reproductive female and two other adults or subadults (using scan sampling with a 3-min time limit), and all occurrences of long calls and the identification of the callers for up to 10 min after playback. The surveyor recorded the playback distance to the GLT group, the number of calls heard from the observed group and the number of animals observed during the first 5 min and up to 10 min after the playback. Playback started when the selected animals were all visible to the observer. We performed only one trial per day per group to reduce habituation to the stimulus of individual vocalizations.

### Censusing GLTs throughout their geographic range

In 2014, when this survey was completed, we recognized seven MUs. As the resolution of available satellite images improved, our understanding of the number, size and connectivity of MUs also improved. In 2019, we recognize 13 MUs. Likewise, our understanding of the amount of forest in each MU improved over time, and this change impacted our estimates of GLTs in each MU. The distribution of GLTs is restricted to forests below 550 m elevation [12] and GLTs routinely traverse open areas up to 100 m [26]. Thus, when determining the area of each MU we included forest that was below 500 m elevation and less than 100 m from other fragments.

**Fig 2:**
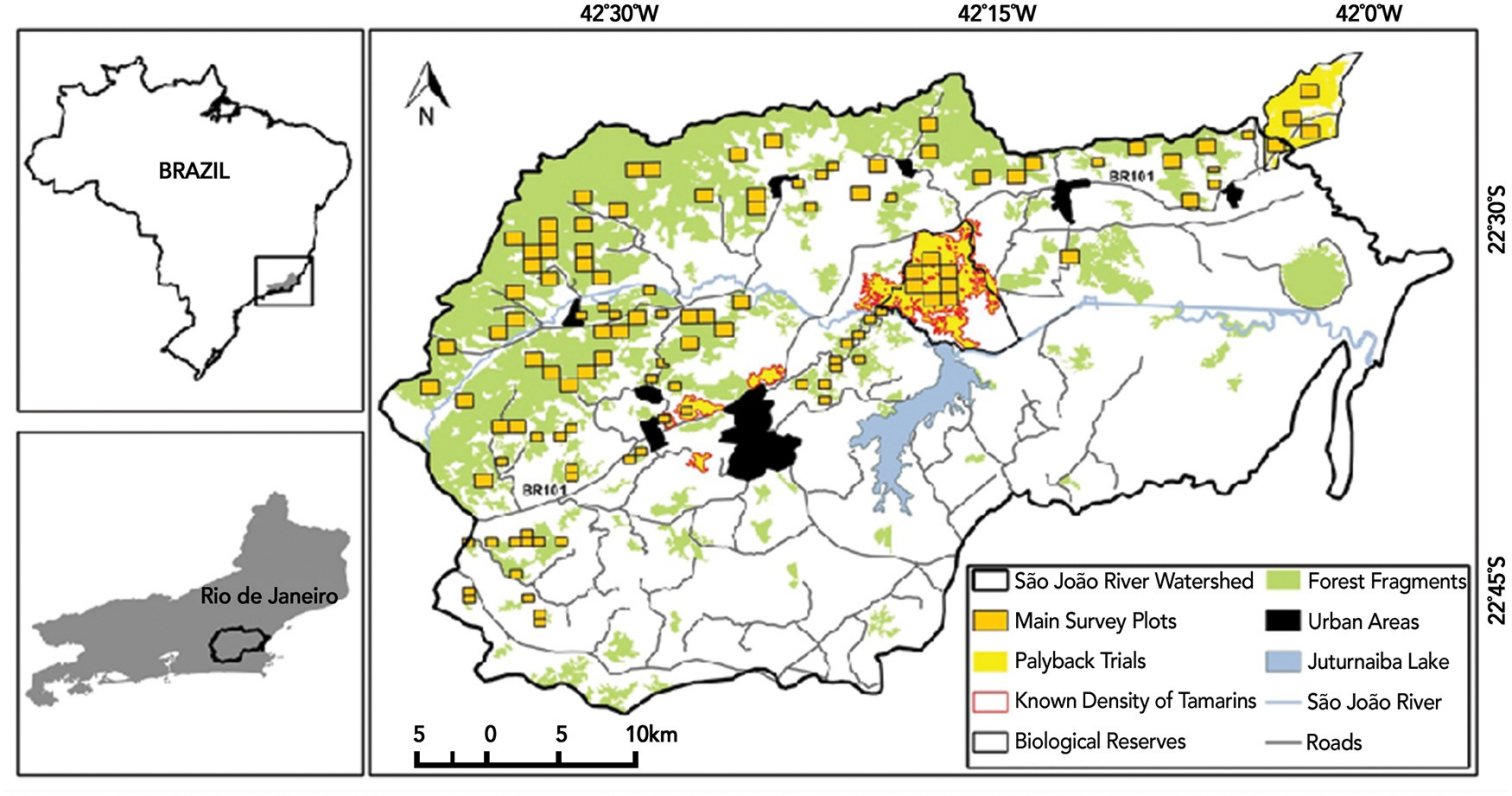
Locations of experimental and survey playbacks in the São João river basin. São João river basin, geographic range of most golden lion tamarins. Forest fragments (green) were identified using Landsat satellite images. Orange squares indicate randomly selected, 48 ha or 120 ha quadrats sampled in the playback survey. Observers played recorded GLT vocalizations at points 200 m apart along transects in each quadrat, noting responses by GLTs. Yellow polygons are locations of experimental playback trials to estimate the detection function. Density of tamarins in fragments bordered by red were obtained from complete counts or home range data.

The forest cover map of the study area was built using a LANDSAT 5 image of the São João River Basin (SJRB) from 2010 [37] and Atlantic Forest images using OLI/LANDSAT 8 at a scale of 1:50,000 in vector format [38]. We used the forest cover map for the study region to calculate the forested area for each MU. All analyses were performed using ArcGIS 10.5. The entire geographic distribution of GLTs was sampled using GLT playbacks between August 2013 and January 2014. We selected areas to be sampled by overlaying a grid of quadrats over a map of the forested area of the watershed and randomly selecting 10% of the quadrats per MU. Quadrats were 48 ha for forest fragments <10 km^2^ or 120 ha for fragments ≥10 km^2^. The objective was to sample 20% of the area of each MU (Fig 2).

The protocol for point transect with lures sampling consisted of two observers visiting each selected quadrat, sequentially walking two pre-determined parallel north-south transects 200 m apart, emitting the playbacks of tamarin long calls at points 200 m apart along the transect (10 points in large quadrats, 6 points in small quadrats), and recording the presence (yes or no) and the number of tamarins observed. The sampling was done between 0700–1100 h. The lure consisted of playing recorded long calls of an adult male and an adult female tamarin (same as those used for the detection experiment), four times in a row, each time with the speaker facing a different cardinal direction. These series were repeated twice at 5 min intervals (total time per point = 10 min). The observers recorded the number of tamarins seen, including sex and age categories. When they could not see the tamarins but could hear them, one of the observers left the transect and approached the tamarins to attempt sightings. At the first GLT detection in a quadrat the observers stopped sampling, moved to the next quadrat and began sampling there.

### Statistical analyses

All statistical analyses were completed in R (v2.12.0). The detection function was calculated using a logistic regression. Distances, group size, categorical fragment size (Large ≥ 10 km^2^ < Small) and group ID were included as covariates. We compared Akaike Information Criterion (AIC) values for each combination of variables. The detection function was then adjusted using generalized linear models for the binary data collected during the survey using “glmer” in the “lme4” package [39] in R. This function included the random effect of group to correct for the dependence of data caused by repeated measures on each group. We used Z tests to compare vocalizations during baseline vs. playback conditions at each distance. We used Wilcoxon tests (V) to examine differences in GLT detection by the surveyor vs. the observer at the 30, 60, 90 and 120 m playback locations and compare the probability of detection between small and large forest fragments.

The detection function calculated from the playback experiment was used to estimate the area sampled for each playback point. GLT abundance was calculated using the Horvitz-Thompson estimator via the “sampling” package in R [40]. We used a bootstrap to estimate the variance in abundance within a 95% confidence interval. We estimated densities by dividing the number of detections during the main survey by the total area sampled in each forest fragment. We compared our results with known densities obtained from complete counts or home range data from sampled forest fragments (Fig 2). We estimated population size for each of seven MUs separately using the group sizes observed during the survey. To calculate population sizes, we multiplied the group density estimate by the average group sizes and multiplied that number by the forested area of each MU.

### VORTEX modeling to set conservation goals

The biological parameters used in modeling GLT population viability were taken from 18 years of continuous monitoring of 8–13 groups of GLTs in Poço das Antas Biological Reserve [29, 41–44] and are available in S1 Appendix, and [26] and [25]. We used VORTEX to estimate the size of a GLT population with these biological characteristics that would be necessary to achieve 0% probability of extinction and at least 98% retention of genetic diversity over 100 years. VORTEX results indicated that a GLT population of 1,000 would be adequate to meet those demographic and genetic standards [25, 26]. We increased that desired population viability estimate to 2,000 as a buffer against future loss of forest. We used GLT density data from the Poço das Antas field study to estimate the amount of forest that would be occupied by 2,000 GLTs ([29], about 25,000 ha). We used these desired future values to establish a measurable conservation goal: By 2025, at least one GLT population with 0% probability of extinction and at least 98% retention of genetic diversity over 100 years as measured by VORTEX modeling. We developed a simple version for communication to the public: By 2025, at least 2000 GLTs living in at least 25,000 ha of connected and protected Atlantic Forest.

## Results

### Experiment to estimate the detection function

In most cases, GLTs responded to the playback by approaching and emitting long calls. Only two or three individuals from the group, typically the reproductive animals, emitted response vocalizations. The reproductive male and female responded in 44% and 35% of the trials, respectively, other adult males in 30% and other adult females in 7% of the trials. The long call emission rate was not significantly correlated with group size (Spearman r = 0.11, p = 0.50). The distance between the playback and the GLT group did not affect the proportion of animals that responded to the playback by approaching the speakers (F = 0.13; df = 3,27; p = 0.9) or that vocalized after the playback (F = 0.49; df = 3,27; p = 0.7). Tamarins called significantly more in response to the playback than during baseline (i.e., no playbacks) at 30 m (Z = 3.01, p = 0.001), 60 m (Z = 1.98, p = 0.05), 90 m (Z = 3.32, p = 0.05) but not at 120 m (Z = 1.41, p = 0.16). The number of long calls heard by the surveyor was significantly fewer than those observed at playback distances of 90 m (V = 96.12, p = 0.01) and 120 m (V = 70.63, p = 0.01), but not at 30 m (V = 22.12, p = 0.09) or 60 m (V = 13.63, p = 0.68).

Several detection function models were fitted to the data from trials using all combinations of available covariates. We used values of the Akaike Information Criterion, AIC, to select the best model: probability of detection is a function of distance from the point and fragment size category (Table 1).

**Table 1:** Model selection criteria.

Variables Included in the Model	df	AIC	delta	weight
**Intercept, Distance, Fragment Size**	**3**	**45.3**	**0.00**	**0.655**
Intercept, Distance, Fragment Size, Group Size	4	47.8	2.46	0.192

The top two ranked logistic regression models (*n* = 40) for the playback trial data with the highest ranked model in bold. Distance is distance from the animal to the playback point (meters), Fragment Size is the binary size category of the sampled forest fragments (larger or smaller than 60 ha), Group Size is the number of individuals in the GLT groups.

**Fig 3:**
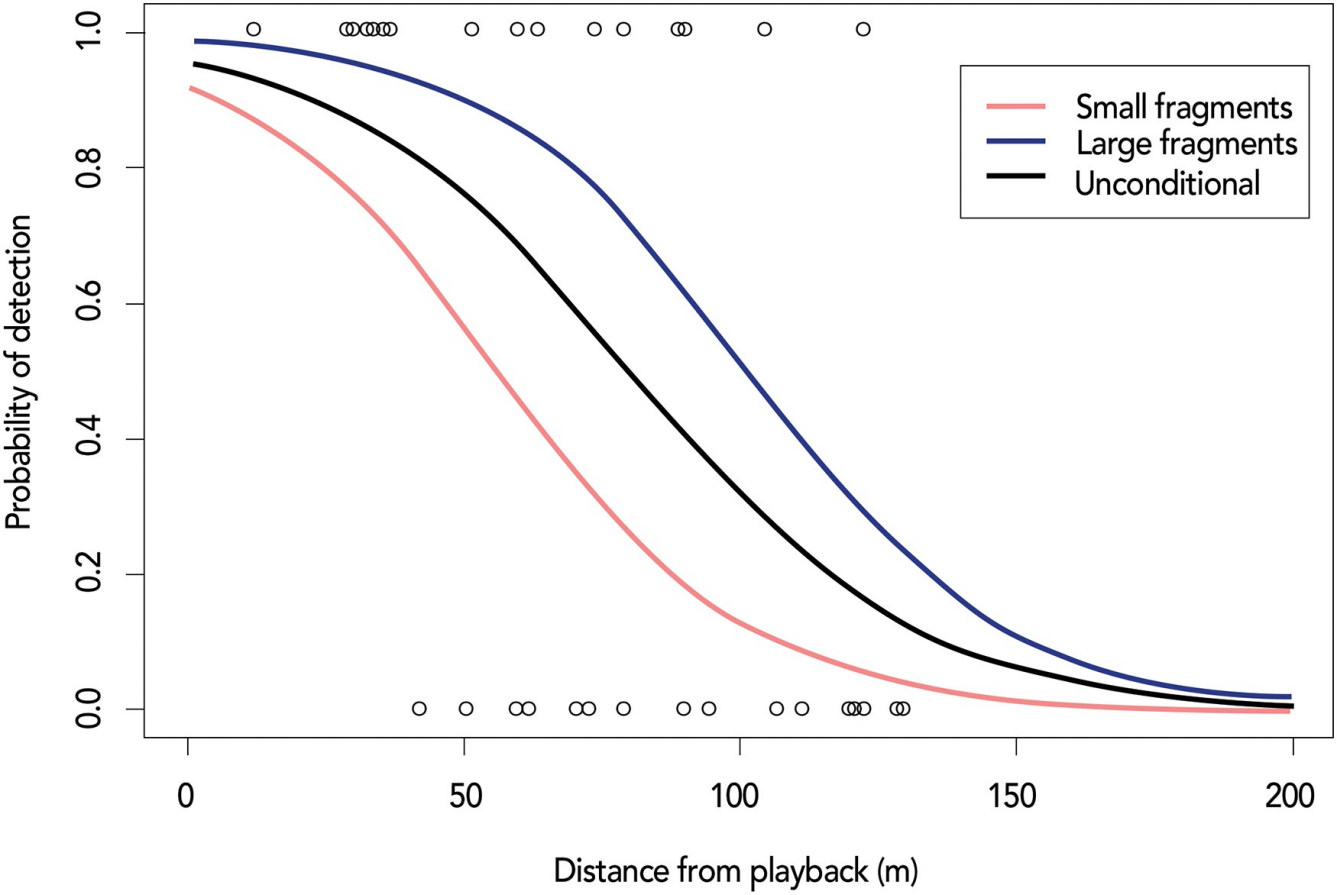
Plot of response against distance of GLT group from the playback point for 40 trials. The range of values along the Y-axis, 1 and 0, represent probability of detecting GLTs during the playback trial. Also shown is the estimated probability of detection as a function of distance from the playback point (black line), and the estimated probability of detection as a function of distance from the playback point conditional to the fragment size (Small fragment (< 60 ha) = red line; Large fragment (3,606 ha, 5,584 ha) = blue line).

The fitted model with probability of detection and distance for different fragment sizes is illustrated in Fig 3; the model’s residual deviance is 38.7 with 38 degrees of freedom, indicating a good fit. We selected a truncation distance of *w* = 200 m, beyond which probability of a response was deemed to be zero; this equates to an effective area surveyed around each point of about 0.03 km^2^ in small fragments and 0.04 km^2^ in large fragments. Estimated probability of detection in large fragments (median = 0.80) was higher than in small fragments (median = 0.37) (V = 21, p = 0.03).

### Results of survey

We surveyed 184 points in small fragments, with 24 social groups detected, and 326 points in large fragments with 38 social groups detected (S1 Dataset). Application of the estimated effective area surveyed to these detections gave densities of 2.6 groups/km^2^ in large fragments and 4.4 groups/km^2^ in small fragments. Corresponding bootstrap 95% confidence intervals based on 3,999 resamples were 1.8–3.6 groups/km^2^ for large fragments and 2.7–6 groups/km^2^ for small fragments. These density estimates were similar to known densities from Poço das Antas Reserve, 1987–2005: 1.6–2.8 groups/km^2^, with an average of 2.25 groups/km^2^ [29]. The observed group densities were multiplied by the average group size observed during the surveys for each point with detections (Table 2). Using this method, we estimate the total number of GLTs in the wild was 3,706 (CI: 1,863–5,383) individuals in 2014. Of these, we estimate that approximately 59% (2,176) are tamarins from original wild populations, 34% (1,281) are descendants from the captive-born reintroduced population and 7.0% (249) are descendants of the translocated groups.

**Table 2:** Results of the playback survey for each management unit.

Management Unit	Group D	Individual D	Group Size	Forest Area	Pop Size	95% CI	Management Type
Aldeia Velha	2.41	7.1	5.06	6,993	499	248–749	Reintroduced
PDA-Cambucaes	2.26	16.2	7.18	4,503	731	570–892	Wild
União-Faz Dourada	1.35	6.1	4.48	4,112	249	102–396	Translocated
Pirineus	1.88	9.7	5.16	13,444	1303	821–1,783	Wild
Rio Vermelho	2.49	19.0	7.64	996	189	154–225	Reintroduced
Imbaú	2.34	19.0	8.12	3,120	593	482–705	Reintroduced
Gaviões-Lavras	0.37	1.7	4.67	8,243	142	36–437	Wild
**Total**	**1.67**	**8.6**	**5.16**	**41,411**	**3,706**	**2,415–5,187**	

Results from the 2014 point transect with lures survey using playbacks of GLT vocalizations. Group D = group density, Individual D = individual tamarin density, Group size is the median observed group size, Forest area is the total area (hectares) of suitable forest in the management unit, Pop. size is the estimated GLT population size in 2014. In the heading, group density is given in groups/km^2^ and individual density is individuals/km^2^. Management Type indicates whether the GLTs were native to the MU, or descendants of GLTs reintroduced or translocated by AMLD. PDA is Poço das Antas Biological Reserve.

### Estimating GLT population viability

The results of VORTEX modeling (Table 3) using population estimates from the playback survey presented in this report indicate that GLTs in all MUs, if managed as a metapopulation, would meet the desired future viability values of 0% probability of extinction with retention of over 98% of its genetic diversity during a 100 yr period. The analyses of seven individual MUs show that all meet the desired future value for the demographic indicator, but only one, in bold, also meets the desired future value for retention of genetic diversity indicator. With an estimated 1,303 GLTs and 13,444 ha of forest that MU alone does not meet AMLD’s conservation goal for the species: 2,000 GLTs in 25,000 ha of connected forest.

**Table 3:** Results of VORTEX GLT population viability modeling.

GLT Population	Starting Popn. Size	Prob. Extinct	N-Extant Population	Gene Div (GD)	SD (GD)
Aldeia Velha	499	0	455.15	96.67	0.5
PDA	731	0	674.78	97.71	0.3
União-Dourada	249	0	222.64	93.4	1.32
Pirineus	1,303	0	1,194.63	**98.69**	0.15
Rio Vermelho	189	0	166.33	91.28	2.14
Imbau	593	0	547.73	97.16	0.47
Gaviões	142	0	123.47	88.58	3.1
Total Metapop	3,706	0	3,384.73	99.54	0

Results from VORTEX modeling of GLT populations in seven MUs and for the total metapopulation. Prob Extinct is probability of extinction calculated as the proportion of iterations that went extinct, N-Extant Population is the mean size of modeled populations surviving to 100 yrs, Gene Div is mean percentage of expected heterozygosity remaining in the extant population with standard deviation (SD) across iterations. PDA is Poço das Antas Biological Reserve.

## Discussion

### Evaluating progress in GLT conservation: Estimating GLT population size

The data from the 2014 survey serve as the baseline for comparison with the results of future playback surveys to evaluate progress toward AMLD’s 2025 goal. Before the results presented in this report were available, AMLD estimated the number of GLTs in the wild by multiplying the minimum GLT density observed in the long-term study in Poço das Antas Reserve [29] by the area of suitable forest in each MU. Estimates of forest fragment area and connectivity improved with access to higher-resolution satellite images. In the current study we report population estimates calculated using a modified point transect with lures method [30] in which lures were playbacks of recorded GLT vocalizations (playback method). Total population estimates using these two methods are 3,200 and 3,706 GLTs, respectively. While these estimates are similar, the survey done using point transect with lures is a preferable method for several reasons. First, GLT density in Poço das Antas varied widely over time, from .07 to .14 GLTs/ha during that 18 yr study [29]. Extrapolation using, for example, mean GLT density for Poço das Antas to all other MUs would yield a much larger estimate than results presented in this study. The current study permits calculation of population sizes for each MU. Second, continuous observation of 8–13 groups of GLTs for nearly two decades in Poço das Antas was relatively lengthy and expensive compared to the 15 months of field work required for the playback experiment and survey. Third, assumptions for the extrapolation method included that GLTs occupied all available forest in the other MUs, as they did in Poço das Antas Reserve, and that GLT densities in the fragments were similar to those in Poço das Antas. The point transect with lures method allowed us to test those assumptions. These concerns notwithstanding, the GLT groups habituated to observers in Poço das Antas and other fragments made possible the experiment necessary to calculate the detection function used in the larger survey.

These results also show the contributions of the three management strategies (conservation of original wild populations, reintroductions of captive-born animals and translocations of wild animals) to the current population. The reintroduction of 153 animals were carried out on private properties and were possible due to the substantial participation of the land owners in the conservation project [11, 15]. Consequently, previously empty forest fragments, many of which were located between separated wild populations, were soon occupied by reintroduced tamarins and later by their descendants (currently 1,281 individuals). The reintroductions and translocations added genetic diversity to the populations living within the management units [26, 45, 46]. Molecular genetic studies of these populations indicate a need to augment gene flow among management units to avoid loss of genetic diversity over time [47, 48].

### Strategic planning to enhance conservation success for golden lion tamarins

**Fig 4:**
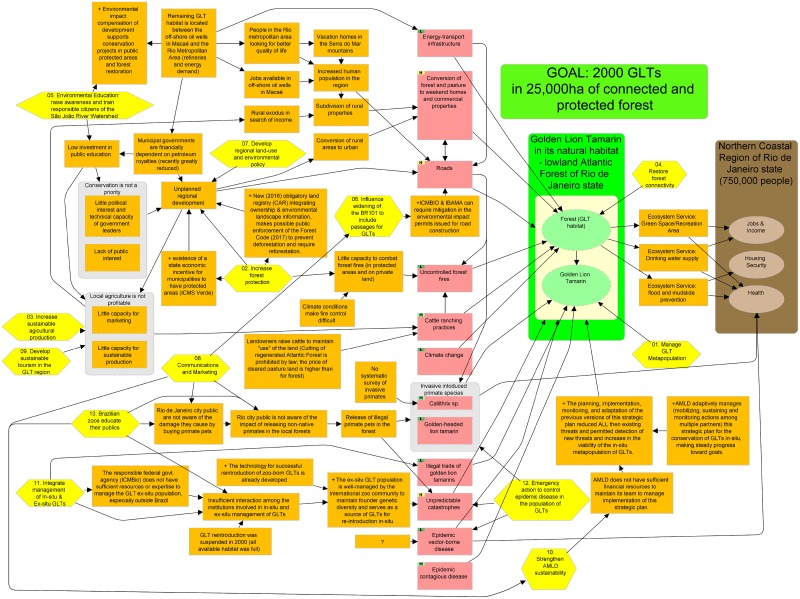
Conceptual model of the 2016–2025 strategic plan for conservation of golden lion tamarins (Version 18 October 2018, translated from Portuguese). Conceptual model showing elements of AMLD’s strategic plan to achieve a viable population of GLTs including the project scope (green rectangle) containing two conservation targets (ovals) and AMLD’s desired conservation outcome (blue rectangle), direct human-caused threats (purple rectangles), contributing factors such as indirect threats or opportunities (small brown rectangles), strategies with actions that have a common focus (yellow hexagons), and benefits to humans that result from GLT conservation (large brown rectangle). Arrows represent presumed causal relationships. The diagram was produced using Miradi project management software.

AMLD’s strategic plan to achieve its goals is illustrated in Fig 4 a conceptual model that depicts the context within which the project operates including the major forces that influence the viability of golden lion tamarins and the connection and protection of forest in the geographic scope [49]. The analysis outlined in the conceptual model was completed with the participation of many experts including researchers, wildlife managers, population managers, protected area managers, foresters, educators, agricultural extensionists, small and large landowners, national and local government officials, and representatives of the international zoo community. Many of the participants have been engaged in golden lion tamarin conservation for two or three decades. AMLD, its predecessor organization, and partners have been conducting this type of situation analysis and adaptive management for conservation of GLTs since 1983.

The geographic scope of AMLD’s program is the natural habitat of the GLT species, the remaining lowland Atlantic Forest of Rio de Janeiro state, Brazil–roughly corresponding to forest under 500 m elevation in the São João river watershed. Conservation of forest habitat for GLTs also provides ecosystem services including green space, drinking water, flood and mudslide protection that contribute to health, housing security, jobs and income to more than 750,000 people living in and beyond the watershed. We identified 11 human-caused threats to GLTs and/or forest and we assigned the highest rank to three threats. We assigned this rank to “conversion of forest and pasture to weekend homes and commercial properties” and “roads” because of the growing portion of forest they affect, the difficulty in restoring forest connectivity once these are in place, and the additional threats they bring to the region. We also rated “unpredictable catastrophes” as a high-level threat because the GLT population is endemic to one small area. One catastrophic event such as a storm or disease could eradicate the entire in-situ population. For each threat we identified indirect threats (i.e. causes), underlying causes and other factors.

To illustrate the logic flow in the conceptual model, following is an explanation of the relationship between one threat, roads, its underlying causes and other factors. Roads are a threat because they reduce and fragment remaining habitat. More roads are being constructed because the human population of the region is growing. It is growing because people in the city of Rio de Janeiro are looking for better quality of life and moving to the geographic scope or building vacation homes there. People are also attracted to the area because of jobs available in the nearby offshore oil-production industry. The remaining GLT habitat is located between offshore oil wells and the city of Rio de Janeiro, which causes an additional threat: gas and oil pipelines, and high-tension electric lines that cross the area and limit possibilities for restoring forest connectivity. The municipal governments in the region are financially dependent on royalties from the energy transport infrastructure, which leads to unplanned regional development in rural areas and construction of more and wider roads dividing the remaining forest.

After completing the analysis of causes of threats, the team looked for intervention points, factors they could change with feasible actions combined into 12 strategies. Two strategies aim to directly restore the conservation targets, and 10 strategies are designed to reduce the threats to the targets. For each of these strategies AMLD used Miradi software to develop a separate results chain diagram [49] (not included in this report) depicting their assumptions about how the strategy will restore the targets or reduce threats, as well as action and monitoring plans that include activities, measurable objectives, indicators and budgets. Each year AMLD reviews the success of these strategies (implementation, assumptions, and impact on achieving a viable population of GLTs) and makes adaptations to the entire strategic plan, including the situation analysis and the definition of its goal. A brief summary of the strategies in AMLD’s strategic plan follows. Strategies are numbered to match those in the conceptual model (Fig 4).

#### 1. GLT metapopulation monitoring and management

Focused directly on restoring the GLT metapopulation to viability, this strategy includes field activities to scientifically manage GLT populations in small, isolated habitat fragments, collection of data on GLT biology and status (such as the survey described in this paper), and detection of and reaction to potential threats to the GLT population, e.g. epizootic diseases. AMLD’s database contains information on ca. 15 groups of GLTs and their habitat collected continuously over 36 years. This information guides our conservation work and informs about our progress.

#### 2. Forest protection

This strategy seeks to permanently protect GLT habitat from the threats of development and further fragmentation by roads and energy-transport infrastructure. We define “protected forest” as areas that meet three criteria: 1. have permanent legal protection; 2. are covered with mature-stature forest or are in the process of restoration—which takes about 7 yrs; 3. have an effective management program. This strategy includes activities to increase the area, forest cover, and effective management of three types of legally protected land in the geographic scope: public conservation units (2 biological reserves, 1 state and 3 municipal parks); 43 permanent private reserves; and areas of private land where permanent forest cover is required under Brazil’s new Forest Code (Lei 12.651/2012). Rural landowners are required to register these areas in the “Cadastro Ambiental Rural” (CAR), a public online land registry that integrates forest cover and land ownership information. The Forest Code requires that rural landowners maintain forest cover on steep slopes and along watercourses, and on 20% of their property. AMLD uses CAR data and satellite image analysis to monitor compliance of the more than 600 privately owned rural properties in the region and to identify properties in which forest restoration is legally required and that will also increase forest connectivity. To date, 29,548 ha of forest is protected in parks, biological reserves and private reserves in AMLD’s geographic scope.

#### 3. Strengthen sustainable agriculture on family farms

This strategy seeks to empower farm families to generate “forest-friendly” income and thus not sell their land to developers. Sustainable agricultural practices also reduce improper use of fire, one of the main threats to GLT habitat. To accomplish this, AMLD works in partnership with government and academic institutions to provide technical support to local agroforestry initiatives and to family run tree nurseries that raise and sell native tree seedlings for use in reforestation. AMLD promotes and provides training in agroecological farming practices and maintains a database of rural landowner properties and conservation actions.

#### 4. Forest restoration

This strategy aims to restore connections between isolated fragments of GLT habitat and to increase the forest cover of legally protected land. This is accomplished through: analysis of existing forest connections and priorities for restoration; developing partnerships with landowners to restore forest in compliance with legal requirements of the Forest Code in areas that are also critical for connecting GLT habitat fragments; developing partnerships with corporations required to finance forest restoration to mitigate their environmental impact; empowering local nursery owners to produce and sell ca. 75 species of native tree seedlings thus ensuring a supply chain in the region; planting forest corridors and restoring degraded areas in public protected areas; monitoring the progress of areas in the process of restoration, and monitoring the overall forest cover and connectivity in AMLD’s geographic scope. To date, AMLD has planted 25 forest corridors and reforested 338 ha.

#### 5. Environmental education—Building awareness and citizenship

This strategy aims to reduce threats caused by unplanned regional development, as well as to increase local public engagement in the restoration and long-term protection of a connected forest landscape. Activities are designed to help local residents learn about the local forest ecosystem (including GLTs), become aware of the benefits it provides to their own well-being, understand the threats to forests, and become engaged in local forest conservation actions. AMLD’s 10-session in-service program Rediscovering the Atlantic Forest (RAF) trains an annual cohort of local teachers and community leaders to integrate these concepts into their teaching and other activities, and thus multiply the number of engaged citizens. AMLD uses social media groups to network RAF alumni (to date, a total of 180 in 8 cohorts) and to support and monitor their continued activities in schools and communities. In 2018, AMLD initiated a new initiative, Guardians of the Forest, to engage adolescents living in rural communities adjacent to priority areas for connecting fragments of GLT habitat. Participants connect with nature and GLTs through experiences designed to inspire, engage, and encourage new discoveries and understandings. AMLD also involves local residents in educational activities conducted at the Education Center at Poço das Antas Reserve, in forests on private reserves and at events conducted in local communities.

#### 6. Influence widening of BR101 highway to include bridges for GLTs

Widening interstate highway BR101 threatens to permanently isolate three large forest fragments and their GLT populations (ca. 732 individuals) from fragments north of the highway. This separation would make it much more difficult for AMLD to achieve a viable population of GLTs. Forested overpasses that GLTs can use are the only effective solution. This strategy focuses on the opportunity presented by a federal law requiring environmental permits for infrastructure construction. Beginning in 2012, AMLD convened meetings, organized an international public petition and informed the many players involved in this issue: local, state, and federal offices of two federal government environment agencies, the federal transportation agency, the federal judicial system, the Brazilian company holding the highway concession, and its multinational shareholders. AMLD convinced federal agencies of the need for forested wildlife bridges over BR101 and that stipulation was included in the construction permit. In November 2018, following 7 years of negotiation and litigation by AMLD, construction began on Brazil’s first forested wildlife overpass. The bridge is designed to connect wildlife populations of Poço das Antas Reserve with those in large forest fragments north of the highway. After completion, AMLD will monitor use of the bridge by GLTs and make recommendations for any adaptations that are deemed necessary.

#### 7. Public policy

This strategy aims to influence the development and implementation of municipal, regional and national public policies that contribute to forest protection and connectivity in AMLD’s geographic scope, and restoration of a viable population of GLTs. Activities are designed to empower institutions in environmental planning, management, and oversight; ensure communication among government and non-governmental institutions involved in environmental management; and communicate details of public policies. AMLD participates in public policy forums and advisory councils for public protected areas, watersheds, endangered species, tourism, land use planning, and contagious-disease control.

#### 8. Communications and marketing

This strategy uses mass media, websites, electronic newsletters, social media, technical publications, conferences, promotional materials, and face-to-face events to reach local, national, and international publics with messages designed to increase support for all of AMLD’s strategies.

#### 9. Regional sustainable tourism

Sustainable tourism addresses threats caused by conversion of rural areas to urban areas by providing forest-friendly income to participating landowners and to the region. Sustainable tourism supports AMLD’s institutional sustainability, provides opportunities for visitors to connect with nature and become engaged in local conservation, and builds local pride in the region’s biodiversity and rural culture. AMLD initiated this strategy with visits to see GLTs in the forest and has since added meals and lodging at partnering family farms, visits to local agroforests, and planting tree seedlings in forest corridors. AMLD works with partners to develop sustainable tourism as a major economic alternative in a region which is one of the economically poorest in the state of Rio de Janeiro, but also the richest in biodiversity.

#### 10. Strengthen AMLD’s institutional sustainability

Because GLT habitat is relatively small and fragmented, and under high pressure for development, the monitoring, management and protection of the species and its habitat will be necessary for the foreseeable future. With a dedicated and competent local staff and a large number of respected collaborators, AMLD is the only organization with the necessary commitment and capacity to coordinate the development, implementation, monitoring, and adaptation of a strategic plan to ensure a viable population of GLTs is achieved and maintained. This strategy focuses on building and maintaining AMLD’s capacity to implement all steps in its strategic plan. If it is to survive on the long term, AMLD must continually invest in improving all aspects of its administration: strategic, technical, financial planning, accounting, fundraising, personnel management, partnerships and communications.

#### 11. Integrate management of the ex situ and in situ GLT populations

This strategy aims to improve the flow of information and support among the 150 zoos holding GLTs worldwide, Brazilian federal agencies and AMLD to ensure that a scientifically managed ex-situ GLT population exists to rebuild the in-situ population after any potential catastrophe and also to prevent the return of the GLT pet trade. Participants in the design and implementation of this strategy include: AMLD staff; Save the Golden Lion Tamarin board members; the international studbook keeper and regional coordinators (North America, Europe, Brazil) who manage the ex-situ GLT populations; representatives of the European zoo association (EAZA) and the IUCN Conservation Planning Specialist Group; and Brazilian government officials responsible for the National Action Plans for Endangered Primates. This strategy’s activities include: genetic and demographic management of the ex-situ GLT population; development of Brazilian government policies and agreements for the management of ex-situ GLT populations located in North America, Europe and Brazil; capacity building for Brazilian zoo educators and animal care staff; and providing the zoo community worldwide with updated information about in-situ GLT conservation.

#### 12. Emergency plan to reduce effects of epizootic disease in the GLT population

In January 2017, dead and dying monkeys were reported in states adjacent to Rio de Janeiro. The cause was yellow fever, a mosquito-borne virus that infects humans and nonhuman primates [50]. Mortality in nonhuman primates is high in some species and serves as an indicator of yellow fever presence, thereby signaling the need for mass vaccination of the local human population. In 2018, AMLD worked with Brazilian human health and environment authorities, yellow fever specialists, and field researchers to develop and begin implementation of an emergency action plan to reduce the impact of yellow fever on GLTs as well as the humans living and visiting the area. The plan includes: collaboration with local health officials to ensure that all people in the region are vaccinated for yellow fever; an awareness campaign to protect people and monkeys in the area; a census of GLT populations to estimate losses to yellow fever; and collaboration with partners to develop a safe and effective yellow fever vaccine for GLTs.

#### Detecting new threats and adapting strategies

The threats to achieving conservation goals for GLTs have changed significantly over the life of the conservation program, thus underlining the importance of monitoring indicators of the viability of the GLT population to detect new threats and evaluate their impact. For example, in 2017, a major outbreak of yellow fever occurred within the current GLT species range [51]. Yellow fever is a vector-borne disease transmitted by mosquitoes. In AMLD’s 2017 threat evaluation vector-borne disease was rated as a low-level threat. As we learn more about its impact on GLTs through the monitoring of the indicators for population size, AMLD may adjust the threat rating accordingly and adapt their strategies and reallocate resources to reduce this new threat. The survey results presented in this report serve as a baseline to evaluate changes in GLT population sizes resulting from this disease or other factors. Modified point transect with lures is a relatively cost-effective method to estimate those changes in population size.

The results presented in this report indicate that the total number of GLTs (ca. 3,706) and amount of forest (41,411 ha) in the geographic distribution of GLTs exceeded values necessary to achieve AMLD’s conservation goal of 2,000 GLTs in connected and protected Atlantic Forest. However, the wide range of GLT densities reported herein suggests that the amount of connected forest necessary to hold a target population of 2,000 GLTs may depend on location of forest fragments. In previous strategic planning we identified 25,000 ha of connected forest as adequate to meet that goal. That amount of connected forest was calculated using a density of 0.08GLT/ha, which was derived from long-term studies in Poço das Antas Reserve [29]. Results from the current study suggest that the amount of connected forest required to hold 2,000 GLTs may be significantly larger or smaller than 25,000 ha, depending on which MUs are connected using planted forest corridors.

## Supporting information

S1 Appendix Baseline data used in VORTEX modeling. (DOCX)

S1 Dataset GPS points of sampled quadrats with data on GLT detections. (XLSX)
