# Supplementary material for: Estimating population sizes to evaluate progress in conservation of endangered golden lion tamarins (Leontopithecus rosalia)
Source: PLoS One. 2019 Jun 5;14(6):e0216664. doi: 10.1371/journal.pone.0216664 (PMC6550383; doi:10.1371/journal.pone.0216664)
Supplement: S1 Appendix — (DOCX) [file pone.0216664.s001.docx]

**S1 Appendix. Information on VORTEX modeling of GLT populations.**

**Table A. Baseline parameters for GLT populations used in VORTEX modeling**.

| ***VORTEX* Parameter** | **Input** |
| --- | --- |
| Number of iterations | 500 |
| Number of years | 100 |
| Extinction definition | 1 sex remains |
| Inbreeding depression | Yes |
| Lethal equivalents (LE) | 4.07 |
| LE subject to purging | 50% |
|  |  |
| *Reproductive Systems* (Long-term Monogamy) | |
| Age of first offspring- males | 4 |
| Age of first offspring- females | 4 |
| Maximum age of reproduction | 16 |
| Maximum number of progeny per year | 5 |
| Frequency of 1 offspring | 21% |
| Frequency of 2 offspring | 58% |
| Frequency of 3 offspring | 8% |
| Frequency of 4 offspring | 12% |
| Frequency of 5 offspring | 1% |
| Density-dependent reproduction | No |
| Percent adult females breeding | 73% (Standard Dev. = 9.4%) |
| Percent adult males in breeding pool | 100% |
| Sex ratio | 1:1 |

**Calculating mortality rates**: As females were expected to have higher mortality during dispersal than males due to their greater difficulty in joining existing groups, the functions used in VORTEX to implement the different mortality rates, as functions of N/K, differed for males and females.

For Females: mortality = M_O_+((N>(K/2))*((M_D_-M_O_)/((K/2)+1))*(N-((K/2)+1)))

For Males: mortality = M_O_+((N>(K/2))*(0.75*(M_D_-M_O_)/((K/2)+1))*(N-((K/2)+1)))

In these formulas K is the population size at carrying capacity; N is the initial population size; M_O_ is the mortality rate at low density (N<0.5K) = 100% mortality events + 50% dispersal events; M_D_ is the mortality rate at high density (N ≥ 0.5K), modeled as an increasing linear function, from M_O_ when N = 0.5 K to a maximum of M_D_ when N = K. The age and sex specific values for M_D_ are shown in Table B in S1 Appendix. These functions were not applied to the first age class since those mortality rates were not affected by dispersal and were significantly different from those of the other age classes.

**Table B. Mortality values for M_O_ and M_D_ used in formulas to model density dependent mortality rates.**

| **Sex** | **Age (Years)** | **M_O_** | **M_D_** | **SD** |
| --- | --- | --- | --- | --- |
| Females | 0 | 32.8% |  | 0.087 |
|  | 1 | 12.0% | 19.6% | 0.133 |
|  | 2 | 12.8% | 24.5% | 0.076 |
|  | 3 | 15.3% | 21.0% | 0 |
|  | Adult | 8.3% | 12.6% | 0.067 |
| Males | 0 | 29.8% |  | 0.07 |
|  | 1 | 12.6% | 15.9% | 0.099 |
|  | 2 | 15.1% | 19.5% | 0 |
|  | 3 | 13.4% | 15.3% | 0.075 |
|  | Adult | 8.9% | 12.5% | 0.082 |
